# Beyond food swamps and food deserts: exploring urban Australian food retail environment typologies

**DOI:** 10.1017/S136898002200009X

**Published:** 2022-05

**Authors:** Cindy Needham, Claudia Strugnell, Steven Allender, Liliana Orellana

**Affiliations:** 1 Deakin University, Global Obesity Centre, Institute for Health Transformation, Geelong 3220, Australia; 2 Deakin University, Biostatistics Unit, Faculty of Health, Geelong, Australia

**Keywords:** Food retail, Food environment, Community nutrition environment, Food swamp, Food desert

## Abstract

**Objective:**

‘Food deserts’ and ‘food swamps’ are food retail environment typologies associated with unhealthy diet and obesity. The current study aimed to identify more complex food retail environment typologies and examine temporal trends.

**Design:**

Measures of food retail environment accessibility and relative healthy food availability were defined for small areas (SA2s) of Melbourne, Australia, from a census of food outlets operating in 2008, 2012, 2014 and 2016. SA2s were classified into typologies using a two-stage approach: (1) SA2s were sorted into twenty clusters according to accessibility and availability and (2) clusters were grouped using evidence-based thresholds.

**Setting:**

The current study was set in Melbourne, the capital city of the state of Victoria, Australia.

**Subjects:**

Food retail environments in 301 small areas (Statistical Area 2) located in Melbourne in 2008, 2012, 2014 and 2016.

**Results:**

Six typologies were identified based on access (low, moderate and high) and healthy food availability including one where zero food outlets were present. Over the study period, SA2s experienced an overall increase in accessibility and healthiness. Distribution of typologies varied by geographic location and area-level socio-economic position.

**Conclusion:**

Multiple typologies with contrasting access and healthiness measures exist within Melbourne and these continue to change over time, and the majority of SA2s were dominated by the presence of unhealthy relative to healthy outlets, with SA2s experiencing growth and disadvantage having the lowest access and to a greater proportion of unhealthy outlets.

The prevalence of obesity continues to increase worldwide^(1,2)^; and despite recommendations to address major drivers of the obesity epidemic such as the food system, interventions remain largely focused on individual lifestyle changes^(3,4)^. The effectiveness of interventions at the individual level is limited by the obesogenic nature of the food system which does not support communities to make healthy choices, e.g. individuals cannot overcome/overpower the entrenched environmental drivers^(3)^. Analysis of data from member countries of the Organisation for Economic Co-operation and Development (OECD) indicates that it is increased supply and consequent consumption of calories that have contributed directly to the increased global prevalence of obesity^(5,6)^. Per capita caloric supply is estimated by collecting data on food supply, calculating the quantity of foodstuffs produced and imported by a country and distinguishing between foods available for human consumption at the retail level and that for other uses (e.g. stock feed)^(7)^.

The observed increase in caloric supply is at least in part driven by the changing distribution and accessibility of food resources (i.e. number, type and location of food outlets) in the ‘food retail environment’^(3)^. Food retail environments provide physical access to the food available to buy and play a key role in influencing food purchasing and subsequent dietary behaviours and prevalence of people with obesity^(4)^. Little is known about how the food retail environment is changing (i.e. quantity and healthiness of outlets) over time, the exception being a handful of studies set in the United Kingdom, North America and Australia which reported increasing numbers of food retail outlets^(8)^, which varied by type and density across geographic areas^(9)^ and by measures of socio-economic position^(10–13)^. For example, in the United Kingdom, one study identified an 80 % growth in food outlets between 1980 and 2000, with the most dramatic growth observed for takeaways and restaurants^(10)^. A second study reported that the density per 10 000 population (using data from the 2001 United Kingdom Census) of takeaway food outlets in Norfolk (United Kingdom) almost doubled between 1990 and 2008, supermarket density also increasing albeit by a smaller margin (29 %)^(13)^. In the current study, takeaway food outlet density increased at a more rapid rate in deprived areas, indicative of the non-uniform way in which food is retailed across and within communities^(13)^. Similarly, in a sample of neighbourhoods in the Bronx (New York) between 2008 and 2017, the growth in food retail establishments was twice that of the population growth (5·7 %) over the same period, with a significantly larger number of less healthy outlets opening in lower-income areas compared with high-income areas^(12)^. Over a shorter period of 10 months (2016–2017), using a sample of urban streets in the Bronx, modest growth in food retail outlets was observed, with a trend of increasing availability of less healthy compared with healthy food options from within food outlets^(8)^.

While there is growing evidence of the relationship between the food retail environment, dietary behaviours and obesity, strong evidence on the relationship is lacking and limits the development and implementation of healthy food retail environment policies^(14)^. Mixed results across studies are likely a consequence of heterogeneity in methods and measures, as ongoing debate exists as to what aspects of food retail environments are most influential on health^(15–17)^. A large portion of the literature seeks to examine disparities in food access and availability, to understand how this might be related to the disproportionate geographical distribution of people with obesity^(15–17)^. To do this, a common approach in the food retail environment literature is to examine absolute measures of access and availability for a single type of food outlet (i.e. density of supermarkets or fast-food outlets only) as a representation of the food retail environment. For example, the well-known term ‘Food Desert’ first used in the 1990s^(18)^ generally refers to areas with limited access to food retailers (supermarkets or grocery stores in most instances), where residents are restricted by physical and in some cases economic barriers to accessing healthy foods^(19)^. The term has successfully been used by the USA Federal Government to implement the ‘Healthy Food Financing initiative’ (HFFI). The HFFI provides funding to support the establishment of new supermarkets and grocery stores in areas identified as Food Deserts; in this instance defined as a low-income census tract within an urban area where at least 33 % of the population cannot access a supermarket or large grocery store within one mile from home^(20)^.

The use of absolute measures involving only one food outlet type has, however, been critiqued due to its simplistic nature^(15,21)^. Results from earlier research suggest that studies encapsulating a broader range of food outlets are more likely to report associations in the expected direction (e.g. greater availability of healthy outlets associated to lower prevalence of people with overweight/obesity)^(15)^. Recent research suggests using relative measures of the food retail environment (i.e. the relative availability of healthy outlets from the sum of healthy and unhealthy food outlets), return more consistent findings in association with food purchasing and consumption behaviour (i.e. greater proportion of healthier outlets associated with healthier purchasing and lower prevalence of people with obesity) than those using only absolute measures^(22,23)^. Using relative measures, the term ‘Food Swamp’ has emerged to describe unhealthy food retail environments where the density of unhealthy food outlets (i.e. independent takeaways and global fast food chains) is much higher relative to healthy food outlets^(24–26)^. While encapsulating more food outlet types, relative measures of the food retail environment have also been critiqued due to inclusion of only outlets classified healthy or unhealthy, this simplistic classification leading to the exclusion of food outlets that are not clearly healthy or unhealthy; and as a result produce a simplified description of the complex and multidimensional food retail environment^(23,27)^. Excluding a large proportion of food outlets because they are difficult to categorise limits the ability to examine health effects, as it seems logical that less healthy and/or independent specialty stores may play an important role in health outcomes, an aspect missing in earlier studies^(28,29)^.

In an attempt to incorporate all food retail outlets, a recently developed tool by Moayyed et al.^(30)^ classifies all food retail outlets into twenty-four food outlet types and uses a Food Environment Score (FES) to categorise their healthiness on a scale from –10 to +10, with zero included as a possible value in the scale. Using this tool, the healthiness of the food retail environment for a given area (i.e. suburb) is calculated by the sum of all food outlets FES, divided by the total number of outlets^(30)^. While inclusive, the FES provides only a measures of healthiness and does not provide a measure of accessibility (i.e. density of food outlets per population or area) which is an important aspect of consideration. Meyer et al.^(23)^ used Latent Class Analysis to longitudinally examine the neighbourhood food and physical activity environment using measures of accessibility (using 3 km buffers) around participant homes and their association with weight-related outcomes (diet quality, fast-food consumption, BMI and physical activity). This holistic approach identified six neighbourhood classes associated to some obesity-related outcomes. The classes were not restricted by pre-existing classifications.

In the current study, we propose to use a census of the food outlets available in Greater Melbourne conducted at four different time points across eight years to identify trends in the food retail environment in an area experiencing rapid population and urban growth. The current study aimed to:

1. Identify the most prominent food retail environment typologies in small geographical areas in Greater Melbourne, Victoria, based on a diverse set of measures of accessibility to all types of food outlets and relative availability of healthy food outlets.
2. Describe how the prevalence of food retail environment typologies: (a) changed across the study period (2008–2016) and (b) varied according to distance from the Central Business District (CBD) or area-level socio-economic position (SEP).

## Methods

### Study region

The current study is set in Greater Melbourne (hereinafter referred as to ‘Melbourne’) the capital city of the state of Victoria, Australia. Victoria is experiencing the fastest population growth in Australia^(31)^.

### Food retail environment data source and classification

A retrospective census of food outlets was undertaken for 2008, 2012, 2014 and 2016; stores were classified by ‘types’ and ‘healthiness’^(11)^. Retrospective food outlet data (name, type, address) for all food outlets located in Melbourne were extracted from hard copy business directories called the Yellow and White Pages, which publish government and commercial lists of businesses from information provided by telecommunications services^(32)^. Limited virtual ground truthing was performed in 2019 using Google and Google Street View to confirm current operation status and premise type^(11)^. Food outlets were classified into 17 types using an Australian food outlet classification tool (adapted to include an additional food outlet type ‘salad bar/sushi bar’) and allocated a FES representing outlet ‘healthiness’ using a 21-point scoring system ranging between –10 (least healthy) and +10 (most healthy) (see online supplementary material, Supplemental Table 1)^(11,30)^. Store types were collapsed into three groups according to their FES. Then, by type into seven groups of which we included only supermarkets in the current study, given they operate at a larger scale than most other retailers and serve a greater proportion of the Australian population (68 % of food purchases were from supermarkets in 2019)^(33)^, warranting consideration independently as well as a contributor to healthier food retail availability (Table 1; see online supplementary material, Supplemental Table 1).

**Table 1:** Food retail environment: food outlet types and classifications

Food outlet types included within each accessibility measure*
1. Supermarkets: Minor & Major supermarkets.
2. Healthy (Healthiness score range: +5 to +10): Supermarkets, Fruit and greengrocer, Butcher, Fish, Poultry shop, Salad/Sandwich/Sushi bar.
3. Less Healthy (Healthiness score range: –4 to +4): Cafes and Restaurants (Independent and Franchise), Bakers, Delicatessen.
4. Unhealthy (Healthiness score range: –10 to –5): Fast-food, Takeaway independent, Pubs, General stores and Specialty extra.
5. Relative Healthy Food Availability: Healthy food outlets (Supermarkets and Greengrocers) Unhealthy food outlets (Fast-food and Takeaway independent).

Adapted from Needham et al.^(11)^

* Descirptions of each food outlt type are listed in Supplemental Table 1.

### Food retail environment: geographic scale

Food outlet data were summarised at the Statistical Area 2 (SA2) level, which are medium-sized general purpose geographical zones (i.e. suburbs, residential districts) where communities interact together socially and economically^(34,35)^. With an average population of 10 000 people, SA2s are the smallest area for which population Census data are released^(34)^. In 2016, there were 302 SA2s located entirely within the borders of Melbourne. The SA2 ‘Melbourne’ (i.e. the CBD) was excluded due to the fact that food outlets in this area mainly service visitors^(36)^; therefore, 301 SA2’s were included in the analysis. Food outlets were geocoded and then spatially joined to the 2016 SA2 boundaries Shapefile^(34)^ creating a data set that indicated, for the purpose of analysis, which SA2 each food outlet was located in.

### Food retail environment measures

Food outlet data for each SA2 were used to create two dimensions of the food retail environment, 1) healthy food *availability* using the measure of Relative Healthy Food Availability (RHFA) and 2) *accessibility* using four measures referred to conjointly as the Food Retail Accessibility Measures (FRAMs) in the current study. Together, these measures indicate how much healthy food is available in a neighbourhood and how far on average people need to travel to access a range of different food outlet types within their neighbourhood.

### Relative Healthy Food Availability

Relative healthy food availability is increasingly being used as a measure of food retail environment ‘healthiness’^(15)^. In the current study, the RHFA represents the percentage of healthy food outlets *available* relative to the total number of food outlets (healthy plus unhealthy) within each SA2 boundary. To be consistent with previous literature and allow for comparability with former studies using more limited food outlet data, the RHFA only included supermarkets and greengrocers as healthy food outlets and only fast-food and independent takeaway for unhealthy food outlets^(37)^.

### Food retail accessibility measures

Currently, there is no gold standard for measuring access to various types of food outlets. Building on previous work,^(23,26)^ we considered four measures of accessibility: density of ‘supermarkets’, ‘healthy’, ‘less healthy’ (i.e. neither clearly healthy nor unhealthy) and ‘unhealthy’ food outlets. Density of ‘supermarkets’ was included because the largest proportion of food is purchased at these retailers. Accessibility (density) within an SA2 was calculated as the number of outlets in each classification per km^2^. This measure indicates the average distance a person needs to travel within the SA2 to access one of these outlets, under the assumption that population and outlets are uniformly distributed across the SA2^(21,38)^.

### Identification of Food Retail Environment Typologies

A two-stage approach was followed to identify typologies: (1) SA2s were grouped into clusters using a K-means algorithm and (2) clusters were collapsed into typologies guided by the existing research evidence. Over the study period, some of the SA2s had no food outlets identified for some of the study years (*n* 29); therefore, 1175 ‘observations’ (i.e. all SA2s with food outlets over the study period) were included in the cluster analysis described in Stage 1.

#### Stage 1. Unsupervised clustering

The K-means algorithm with Euclidean (L2) distance was used to sort, based on measures of availability and accessibility, 1175 observations into K = 20 mutually exclusive groups^(39)^. K = 20 was chosen to avoid collapsing large clusters, while retaining atypical clusters with few observations. Five measures of the food retail environment (RHFA and the four FRAMs; density of supermarkets, healthy, less healthy and unhealthy outlets per km^2^) were used as input variables. Variables were standardised using robust measures of location (median) and scale (median absolute deviation, MAD). Input variables were summarised by cluster. The cluster analysis was generated using SAS software version 9.4.

#### Stage 2. Evidence-based grouping of clusters in typologies

With the aim of further collapsing similar clusters into a smaller number of typologies, meaningful thresholds for each of the five measures were derived from earlier studies that examined the effect of RHFA and accessibility measures on behaviours (i.e. food purchasing behaviour and diet) and health outcomes (i.e. obesity prevalence). Supplementary file 2 provides supporting information for thresholds.

First, the cluster RHFA mean was classified into three levels (i.e. ≤ 25 %, > 25 to < 50 %, ≥ 50 %). Then, each of the four FRAM means in a cluster was categorised in levels. For healthy, less healthy and unhealthy density we defined: ‘Low’ (< 1 per km^2^), ‘Moderate’ (≥ 1 to < 2 per km^2^) or ‘High’ (≥ 2 per km^2^) as described in Table 2. These FRAM categories reflect ‘access’ as a measure of distance only and do not reflect what would be considered ‘good’ access required for health. For supermarkets, given their larger size and scale of operation^(33)^, we defined access as ‘Low’ (< 0·625 per km^2^), ‘Moderate’ (0·625 to < 1·25 per km^2^) and ‘High’ (≥ 1·25 supermarkets per km^2^). The four dimensions of access were highly correlated; i.e. where the four access measures were low they were all very low (see online supplementary material, Supplemental Table 3, clusters 13 and 8); when one measures was moderate the other measures whilst low were higher in comparison (see online supplementary material, Supplemental Table 3, cluster 15); when one measure was high all others tended to be high or moderate. Therefore, the four access dimensions were summarised in one of three ‘access’ categories (low, moderate and high) based on the categorisation of the FRAMs: ‘Low access’ if all FRAMs were ‘low’; ‘Moderate access’ where at least one (regardless of type) FRAM was ‘Moderate’ and ‘High access’ where at least one (regardless of type) FRAM was ‘High’.

**Table 2:** Thresholds used for the classification of each food retail environment measure

Measures	Categories	Classification
Relative Healthy Food Availability (RHFA)	Percentage of healthy food resources	RHFA
	≤ 25 %	Low
	> 25 to < 50 %	Moderate
	≥ 50	High
Food Retail Accessibility Measures (FRAM)	Density per km2	Access
Healthy, Less Healthy, Unhealthy	< 1	Low
	≥ 1 to < 2	Moderate
	≥ 2	High
Supermarkets	< 0·625	Low
	0·625 to < 1·25	Moderate
	≥ 1·25	High

### Exploratory analysis of typologies by geographical location and socio-economic position

Each SA2 was classified (based on the local government area (LGA) in which they were located) relative to distance from Melbourne’s CBD as: ‘Inner Ring’ (< 15 km), ‘Middle Ring’ (15–25 km) and ‘Outer Ring’ (25–55 km)^(11)^. A fourth group included SA2s located in LGAs identified as Growth Areas (30–70 km from CBD; areas housing a large proportion of urban growth located on the urban fringe)^(40)^.

The Australian Bureau of Statistics Socio-Economic Index for Areas, Index of Relative Socio-Economic Advantage and Disadvantage (SEIFA-IRSAD) at the SA2 level was used to define SEP quartiles, Q1 (lowest SEP) to Q4 (highest SEP). SEIFA-IRSAD incorporates 25 collected measures of SEP (i.e. income, occupation, education, internet connection) which are used to summarise the relative disadvantage of the population within an area^(41–43)^. Food retail environment data for years 2008 and 2012 were matched to the SEIFA-IRSAD quartiles from the 2011 census^(41)^ and 2014 and 2016 food retail environment data to the 2016 census^(42)^. Four SA2s had missing SEIFA-IRSAD due to the low population or low response rate for that census year^(43)^.

We report prevalence of typologies for each time point to explore trends over the study period by geographic location or area-level SEP.

## Results

Six food retail environment typologies were identified for Melbourne SA2s, five using the two-stage procedure and a last typology corresponding to zero food outlets (Table 3).

**Table 3:** Summary of food retail environment measures for each food retail environment typology by year

	2008		2012		2014		2016	
Year	Mean	sd	Mean	sd	Mean	sd	Mean	sd
Typology: Low access – High % healthy								
Number of SA2s (%)	23	7·6	22	7·3	19	6·3	17	5·7
RHFA %	64·3	17·5	65·0	17·3	68·8	19·8	66·3	19·0
Density Healthy per km^2^	0·2	0·5	0·3	0·6	0·4	0·8	0·3	0·8
Density Less Healthy per km^2^	0·3	0·7	0·4	0·9	0·4	0·9	0·3	0·4
Density Unhealthy per km^2^	0·2	0·3	0·1	0·2	0·1	0·2	0·1	0·2
Density Supermarkets per km^2^	0·1	0·2	0·1	0·2	0·1	0·2	0·1	0·9
Typology: Low access – Low % healthy								
Number of SA2s (%)	81	26·9	59	19·6	46	15·3	42	14
RHFA %	3·1	4·9	3·0	5·2	4·2	5·6	3·5	5·1
Density Healthy per km^2^	0·1	0·2	0·1	0·2	0·2	0·2	0·2	0·2
Density Less Healthy per km^2^	0·6	0·6	0·5	0·6	0·7	0·7	0·7	0·7
Density Unhealthy per km^2^	1·0	0·9	0·8	0·8	0·9	0·8	0·9	0·9
Density Supermarkets per km^2^	0·0	0·1	0·0	0·1	0·0	0·1	0·0	0·1
Typology: Moderate access – Low % healthy								
Number of SA2s (%)	85	28·2	99	32·9	101	33·6	99	32·9
RHFA %	22·0	6·9	24·3	6·8	24·9	7·3	24·0	7·6
Density Healthy per km^2^	0·5	0·3	0·5	0·3	0·5	0·3	0·4	0·3
Density Less Healthy per km^2^	0·9	0·7	0·9	0·6	0·8	0·6	0·9	0·7
Density Unhealthy per km^2^	1·2	0·7	1·3	0·8	1·2	0·9	1·2	0·8
Density Supermarkets per km^2^	0·2	0·1	0·2	0·1	0·2	0·1	0·2	0·1
Typology: High Access – Low % healthy								
Number of SA2s	39	13·0	45	15·0	43	14·3	49	16·3
RHFA %	18·1	9·9	19·3	7·8	19·0	8·1	18·9	8·1
Density Healthy per km^2^	1·9	1·3	2·1	1·8	2·4	1·9	2·5	2·3
Density Less Healthy per km^2^	10·5	12·1	11·6	14·8	11·2	13·6	11·9	14·8
Density Unhealthy per km^2^	5·7	4·1	6·1	5·5	6·5	5·7	6·7	5·9
Density Supermarkets per km^2^	0·5	0·5	0·7	0·7	0·7	0·5	0·7	0·6
Typology: High access – Moderate % healthy								
Number of SA2s	59	19·6	69	22·9	88	29·2	90	29·9
RHFA %	32·0	10·6	32·5	8·1	30·9	8·1	31·5	9·6
Density Healthy per km^2^	2·3	1·6	2·4	1·8	2·6	1·9	2·4	1·5
Density Less Healthy per km^2^	7·4	10·7	5·5	5·4	6·2	9·0	5·9	8·2
Density Unhealthy per km^2^	3·8	3·5	3·9	2·6	4·1	3·1	4·1	3·1
Density Supermarkets per km^2^	0·8	0·4	1·0	0·5	1·0	0·5	1·0	0·6
Typology: Zero Food Retail*								
Number of SA2s	22	4·7	15	2·3	12	1·3	12	1·3

* Represents Statistical Area 2’s with zero food retail outlets.

RHFA %: percentage of healthy food outlets relative to healthy and unhealthy food retail outlets within each SA2.

SA2: Statistical Area 2: medium-sized general purpose areas representing geographical areas where community interact together socially and economically^(34)^.

*Typology 1. Low access – High % healthy:* comprised a single cluster of 81 ‘observations’ (SA2s across years) with low food retail accessibility measures and the highest percentage of healthy food outlets. *Typology 2. Low access – Low % healthy:* comprised a single cluster of 228 observations (SA2s across years) with low food retail accessibility measures and the lowest percentage of healthy food outlets. *Typology 3. Moderate access – Low % healthy:* comprised one single cluster comprising 384 observations with moderate accessibility to unhealthy outlets and low accessibility to supermarkets, healthy and less healthy outlets; and a low percentage of healthy outlets. *Typology 4. High Access – Low % healthy:* comprised 10 clusters with a total of 176 observations (SA2s across years) with high access to all outlet types except supermarkets which was moderate in some of the clusters and a low percentage of healthy food outlets. *Typology 5. High Access – Moderate % healthy:* comprised seven clusters which together contained 306 observations (SA2s across years) with high access to all outlets excluding supermarkets for which there was moderate access on average across the clusters and a moderate percentage of healthy outlets. *Typology 6. Zero Food Retail:* included 29 observations (SA2s across years) that had zero food retail outlets.

Over the study period (2008–2016), the food retail environment experienced an increase in RHFA and accessibility to food retail (Table 3). In 2008, the two most dominant typologies were *Low access – Low % healthy* and *Moderate access – Low % healthy*, together accounting for 55·1 % of SA2s. In 2016, *Moderate access – Low % healthy* and *High Access – Moderate % healthy* typologies accounted for 62·8 % of all SA2s. Over time, the proportion of *Zero Food Retail*, *Low access – Low % healthy and Low access – High % healthy* SA2s slightly decreased by 3·4 %, 12·9 % and 1·9 %, respectively. In contrast, an increase of 4·7 % was observed for *Moderate access – Low % healthy* and 3·3 % for *High access – Low % healthy*, with the largest increase (10·3 %) observed in *High Access – Moderate % healthy*.

### Distribution of food retail environment typologies across geographical location and time

Table 4 presents the distribution of food retail environment typologies within years (rows) and within LGA-Ring (columns). The prevalence of typologies representing *High access* decreased when moving away from the CBD (Table 4, rows). This pattern was seen in all four years, although there was a small increase in the proportion of *Moderate access – Low % healthy* typologies in the Growth Area LGA-Ring over the study period (18·8 % to 33·3 %). The Inner and Middle Ring had the highest proportion of SA2s classified as *High Access – Moderate % healthy* (27·1 % and 57·6 %) in 2008, slightly decreasing over time (Table 4, columns). Of all typologies, the proportion of *High Access – Low % healthy* was highest in the Inner Ring in 2008 (51·3 %), decreasing over time with the Middle Ring having the highest proportion (44·9 %) in 2016. In 2008, close to half (48·1 %) of the SA2s in the Growth Ring were classified as *Low access – Low % healthy* and two-thirds (64·3 %) were classified as *Zero food retail*. Over time the proportion of *Moderate access – Low % healthy* SA2s in the Growth Area Ring increased (18·8 % to 33·3 %). Over the study period, the prevalence of SA2s classified as *Zero food retail* decreased across all LGA-Rings except for the Outer Ring where it remained relatively stable. The Middle Ring experienced an increase in the proportion of *High access – Low% Healthy* (35·9 % to 44·9 %) and a decrease in *Moderate access – Low % healthy* (44·7 % to 33·3 %) and *High Access - Moderate % Healthy* (57·6 % to 47·8 %) typologies. Supplemental File 4 presents maps of ‘typologies’ across Melbourne over time by LGA Ring.

**Table 4:** Food retail environment typology prevalence across years and geographic distance from CBD, in Greater Melbourne

							LGA-Ring							
	INNER			MIDDLE			OUTER			GROWTH				
Food Retail Environment Typology	No. SA2s	% within Inner	% within year	No. SA2s	% of within Middle	% within year	No. SA2s	% within Outer	% within year	No. SA2s	% within Growth	% within year	Total SA2s	% of total
Year: 2008														
Zero food retail	1	2·2	7·1	1	0·9	7·1	3	4·3	21·4	9	11·8	64·3	14	4·6
Low access – High % healthy	2	4·4	8·7	3	2·8	13·0	10	14·1	43·5	8	10·5	34·8	23	7·6
Low access – Low % healthy	3	6·7	3·7	19	17·4	23·5	20	28·2	24·7	39	51·3	48·1	81	26·9
Moderate access – Low % healthy	3	6·7	3·5	38	34·9	44·7	28	39·4	32·9	16	21·1	18·8	85	28·2
High access – Low % healthy	20	44·4	51·3	14	12·8	35·9	3	4·2	7·7	2	2·6	5·1	39	13·0
High access – Moderate % healthy	16	35·6	27·1	34	31·2	57·6	7	9·9	11·9	2	2·6	3·4	59	19·6
	45	100·0	14·9	109	100·0	36·2	71	100·0	23·6	76	100·0	25·2	301	100·0
Year: 2012														
Zero food retail	0	0·0	0·00	0	0·0	0·0	3	4·2	42·9	4	5·3	57·1	7	2·3
Low access – High % healthy	2	4·4	9·1	1	0·9	4·5	10	14·1	45·4	9	11·8	40·9	22	7·3
Low access – Low % healthy	4	8·9	6·8	14	12·8	23·7	15	21·1	25·4	26	34·2	44·1	59	19·6
Moderate access – Low % healthy	2	4·4	2·0	37	33·9	37·4	30	42·3	30·3	30	39·5	30·3	99	32·9
High access – Low % healthy	22	48·9	48·9	17	15·6	37·8	4	5·6	8·9	2	2·6	4·4	45	14·9
High access – Moderate % healthy	15	33·3	21·7	40	36·7	58·0	9	12·7	13·0	5	6·6	7·2	69	22·9
	45	100·00	14·9	109	100·0	36·2	71	100·0	23·6	76	100·0	25·2	301	100·0
Year: 2014														
Zero food retail	0	0·0	0·00	0	0·0	0·00	2	2·8	50·0	2	2·6	50·0	4	1·3
Low access – High % healthy	2	4·4	10·5	1	0·9	5·3	11	15·5	57·9	5	6·6	26·3	19	6·3
Low access – Low % healthy	2	4·4	4·3	13	11·9	28·3	12	16·9	26·1	19	25·0	41·3	46	15·3
Moderate access – Low % healthy	4	8·9	4·0	30	27·5	29·7	29	40·9	28·7	38	50·0	37·6	101	33·5
High access – Low % healthy	17	37·8	39·5	18	16·5	41·9	5	7·0	11·6	3	4·0	7·0	43	14·3
High access – Moderate % healthy	20	44·4	22·7	47	43·1	53·4	12	16·9	13·6	9	11·8	10·2	88	29·2
	45	100·0	14·9	109	100·0	36·2	71	100·0	23·6	76	100·0	25·2	301	100·0
Year: 2016														
Zero food retail	0	0·0	0·00	0	0·0	0·0	3	4·2	75·0	1	1·3	25·00	4	1·3
Low access – High % healthy	1	2·2	5·9	1	0·9	5·9	9	12·7	52·9	6	7·9	35·3	17	5·6
Low access – Low % healthy	2	4·4	4·8	10	9·2	23·8	11	15·5	26·2	19	25·0	45·2	42	13·9
Moderate access – Low % healthy	3	6·7	3·0	33	30·3	33·3	30	42·3	30·3	33	43·4	33·3	99	32·9
High access – Low % healthy	21	46·7	36·7	43	39·5	44·9	13	18·3	10·2	13	17·1	8·2	90	29·9
High access – Moderate % healthy	18	40·0	23·3	22	20·2	47·8	5	7·0	14·4	4	5·3	14·4	49	16·3
	45	100·0	14·9	109	100·0	36·2	71	100·0	23·6	76	100·0	25·2	301	100·0

CBD: Central business district; LGA: local government area; SA2: Statistical Area 2; Inner = local government areas surrounding the CBD; Middle = local government areas surrounding the Inner ring; Outer = local government areas surround the Middle ring; Growth = designated areas to house population growth located on the urban fringe.

### Food retail environment typology over time by area-level socio-economic position: Descriptive analysis

**Fig. 1:**
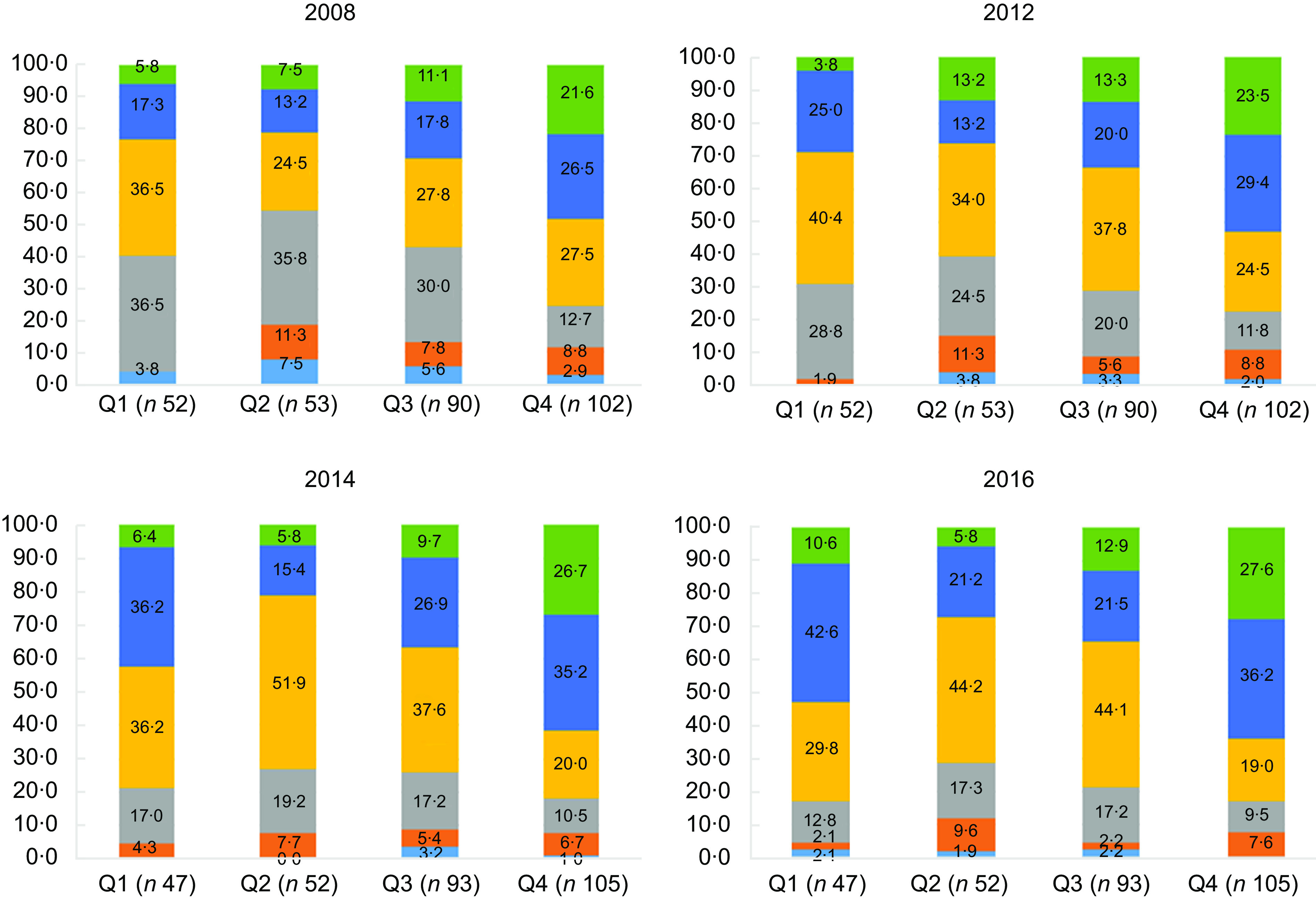
Food retail environment typology distribution by area-level socioeconomic position quartiles within years. SEIFA-IRSAD: Socio-Economic Index for Areas, Index of Relative Socio-Economic Advantage and Disadvantage (Q1 = low socioeconomic position, Q4 = high socioeconomic position). 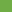, High access – moderate % healthy; 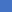, High access – low % healthy 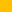, Moderate access – low % healthy 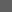, Low access – low % healthy 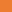, Low access – high % healthy 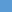, Zero food retail

Figure 1 and Supplemental File 4 present the distribution of ‘typologies’ within SEP (SEIFA-IRSAD) quartiles across Melbourne over time. It should be noted that Melbourne had an over-representation of the second highest (Q3) and highest (Q4) SEP quartiles. SA2 typologies representing RHFA and accessibility were not evenly distributed across SEIFA-IRSAD quartiles. There was a greater prevalence of *High access* typologies in areas of high SEP (Q4) compared to low SEP (Q1). Over time there was an increase in overall accessibility and RHFA across all SEP quartiles (Fig. 1). Supplementary Table 5 presents the distribution of typologies across SEP quartiles within each year (across rows). High SEP SA2s (Q4) housed over half (56·4 %, *n* 22) of all *High access – Low % healthy* SA2s, this amount slightly increased over time. The highest SEP (Q4) SA2s maintained the largest portion of *High access – Moderate % healthy* (45·8 %, *n* 27 in 2008; 42·7 %, *n* 38 in 2016) across the study period. The proportion of *Low access – High % healthy* was highest in high SEP SA2s (Q4), remaining constant over time (range 40·9 %, *n* 9 in 2008; 50 %, *n* 8 in 2016). In 2008 *Low access – Low % healthy* typologies was highest in the second highest SEP SA2s (Q3: 34·6 %, *n* 27) this trend remaining over time. The second lowest SEP SA2s remained relatively stable in the mix of typologies over time, made up by predominantly *Low and Moderate access* typologies.

## Discussion

We identified six distinct food retail environment typologies across the 301 SA2s in Melbourne between 2008 and 2016. All but one had low RHFA (i.e. low availability of healthy food stores relative to the sum of healthy and unhealthy outlets), and all had greater accessibility to unhealthy and less healthy food outlets, when compared with healthy food outlets and supermarkets. Three of the possible combinations of accessibility and availability were not identified in Melbourne: *Moderate access – Moderate % healthy, Moderate access – High % healthy* and *High access – High % healthy*.

The majority of typologies were considered *Low % healthy*, with the average proportion of healthy food outlets available ranging from as low as 3·1 % in SA2’s classified as *Low access – Low % healthy* to 24·9 % in *Moderate access – Low % healthy* SA2s. Considered alongside the estimated population density of each SA2, this reflects approximately two-thirds of Melbourne residents (70 % in 2008; 62 % in 2016) living in SA2s where the food retail environment includes a large majority of unhealthy food outlets. An increase in access to food outlets was observed across Melbourne over time, with prevalence of *Moderate* and *High access* typologies increasing and *Low access* and *Zero* decreasing.

If increasing availability (RHFA) of healthy food and better accessibility were considered an indicator of typology healthiness, the *High Access – Moderate % healthy* typology would be the healthiest, albeit access to unhealthy and less healthy outlets in this typology far exceeded that of access to supermarkets and healthy outlets. Only one typology was identified as *High % healthy* (i.e. greater than 50 % healthy outlets); however, this was associated with a limited overall availability of outlets (e.g. ≤ 0·4 healthy, less healthy, unhealthy outlets; and 0·1 supermarkets per km^2^). The SA2s closest to the CBD (Inner) and in the highest SEP quartile (Q4) were classified predominantly as *High Access* (i.e. not Food Deserts). The lowest SEP quartile (Q1) showed a small increase in *High Access – Moderate % healthy* over the study period.

Outer and Growth Area Rings housed the largest proportion of *Zero*, *Low* and *Moderate Access* typologies. *Moderate access* typologies became more prevalent over time in both Outer and Growth Area LGAs reflective of an increase in access to unhealthy food outlets to approximately 1 per km^2^, while access to all other food outlet types remained ‘Low’.

The current study indicates that the characteristics of the food retail environment are likely heightening the risk of unhealthy dietary behaviours and increasing prevalence of people with obesity in Melbourne^(37)^. The retail mix reflects similar characteristics in the food retail environment to that reported in New Zealand, Canada and parts of the USA, where ‘Food Swamps’ (areas with greater access to unhealthy outlets relative to healthy outlets) dominate the food retail environment^(25,26,44)^. If we were to have used the term ‘Food Swamp’ to define the food retail environments in the current study, all SA2s excluding the *Low Access – High % healthy* SA2s would be considered ‘Food Swamps’, despite their vastly different characteristics. Parts of Melbourne would also be considered ‘Food Deserts’ when applying the United States Department of Agriculture definition, which considers ‘Food Deserts’ areas where at least 33 % of the population (particularly in low-income areas) cannot access a supermarket or large grocery store within one mile (1·6 km) from home^(20)^. This definition includes SA2s with *Low* and *Moderate Access*, as they have limited supermarket access (from 0·1 to 0·2 supermarkets per km^2^), only *High Access* areas would be considered as having sufficient access. Thus, the approach used in the current study to classify the food retail environment highlights the simplistic nature of these two terms (Food Deserts and Swamps), emphasising the need for a more integral analysis of food retail environment measures, to reflect the complexity and multidimensional aspects of food retail environments across areas.

### Health implications – food retail environment research

Evidence suggests that both having poor access to healthy food outlets and high access to unhealthy outlets relative to healthy outlets are associated with unhealthy weight^(17,37,45,46)^. For example, an Australian study set in Adelaide found that one sd increase in the ratio of unhealthy to healthy food outlets within 1 km of participant’s home address was associated with an 11 % higher risk of participants having abdominal obesity^(47)^. Another study among adults (≥ 45 years) in Sydney (Australia) also found significantly higher BMIs where unhealthy outlets accounted for ≥ 25 % of all food outlets within a 1·6 and 3·2 km buffer from home^(37)^. Another study in Perth (Australia) found that with each additional healthy food outlet within 800 m of home, there was a 20 % decrease in the risk of a child being overweight or obese after controlling for SEP, physical activity, sedentary behaviour, takeaway consumption, age and the presence of unhealthy outlets^(46)^. Given the evidence presented in the current study, findings suggest the characteristics of food retail environment in Melbourne are likely increasing the risk of unhealthy diet and weight^(37)^.

### Food retail environment disparities

These findings highlight the inequities that exist within the food retail environment, with communities living in areas of lower SEP and further from metropolitan centres exposed to unhealthier food retail environments^(11,15,19)^. Several studies have found food retail environment disparities and negative health outcomes among lower SEP populations^(48,49)^. Evidence suggesting healthier food retail environments (i.e. areas with greater access to healthy food outlets) within 800 m and 1 km of home are supportive of a healthy BMI in areas of high disadvantage, but not so for those in areas of less disadvantage^(48)^. Similarly, in Melbourne, women without high school degrees or above living in low SEP areas had a higher BMI, partially explained by lower access to supermarkets, the coastline and sports facilities, when compared with women with the same education level in high SEP areas^(50)^.

Food retail environment disparities have also been reported across Melbourne with people residing in Established Areas (urban areas not experiencing significant development and population growth) having significantly lower BMI and greater proximity and access (density) to supermarkets (within 800 m, 1·6 km and 2 km and 3 km) and fast-food (within 800 m, 1 km, 1·6 km, 2 km and 3 km) when compared with people in Growth Areas (i.e. new housing development areas)^(48,51)^. Unexpectedly, further analysis of the data indicated fast-food density was positively associated with BMI in more established areas of Melbourne (within 800 m and 1000 m buffers), but negatively associated in Growth Areas (within 800 m and 1600 m buffers); after adjustment for a number of factors, including supermarket access, age, gender, measures of SEP and food and beverage consumption^(51)^.

It has been postulated in earlier research that the relationship between the food retail environment and obesity or dietary behaviours across areas, based on location and SEP, is driven by having greater access to healthier food outlets (e.g. supermarkets), which may play a protective role for BMI^(15)^. This is exemplified in the USA, where individuals with limited access to public transport and who did not own a vehicle appeared more vulnerable to the negative health impacts of living in areas where access to unhealthy food was disproportionately higher compared with healthy food, even after controlling for measures of SEP, recreation/fitness facilities and food deserts (absence or presence of supermarkets)^(25)^. In these instances, the physical environment in lower density areas (e.g. Growth Areas) where heavy car dependency, poor public transport, lower housing density and higher relative unhealthy food outlet accessibility is evident, we can expect an increased risk of unhealthy weight independent of SEP^(25,51)^.

### Strengths

Our approach to examining the food retail environment extends previous methods to identify new typologies, and our census of food outlets in 301 geographic areas gives a more detailed perspective of the food retail environment over time than has previously been reported. Results highlight the limitations of considering the food retail environment using only absolute measures of a single food outlet type, or use of terms such as Food Swamp and Food Desert, which by definition are simplistic. For example, despite extensive differences across measures of accessibility when considering only RHFA almost the entirety of Melbourne would be classified as a Food Swamp. However, when considering accessibility to healthy food outlets and supermarkets, a large proportion (all bar typologies that identify as *High Access* SA2s) would also be identified as a food desert. Using a combination of a data-driven and evidence-based approaches, we highlight the complexity of the food retail environment that exists across areas. The inclusion of supermarkets, which account for the bulk of food purchases in Australia (68 % of purchases in Australia in 2019)^(33)^, and healthy and unhealthy food retailers, which are the most influential food outlet types on purchasing and dietary behaviours, are strengths of the study^(52)^. The current study also included the often overlooked food outlets that cannot be clearly categorised as healthy or unhealthy, termed ‘less healthy’ food outlets, further strengthening results^(11)^. By measuring accessibility with density of food outlets within a predefined area unit (e.g. postcode, suburb), we proposed a method that can be applied at scale in public health settings^(15,17)^.

### Weaknesses

The food retail environment data set was extracted from hard copy business listings in the Yellow and White Pages, ground truthing was performed in 2019 on a sample of outlets and was limited by its retrospective nature^(11)^. Therefore, caution should be taken when assuming all food outlets are represented, as some outlets (e.g. those without a fixed-line telephone service or business listing) may have been overlooked. Supermarkets are often considered a the major source of fruit and vegetables within the food retail environment^(22)^ and are commonly used as a proxy for healthy food retail outlet availability^(15)^. However, they are also a large retailer of unhealthy food products^(53)^ and have a tendency to promote unhealthy food products heavily instore^(54,55)^. Following the Australian food outlets classification tool (range –10 to +10), supermarkets (as well as salad, sushi and sandwich outlets) received a rating of +5, to reflect their contribution in retailing unhealthy as well as healthy food. Accessibility measures represent the average travel distance within each SA2 to access a food outlet, under the strong assumption that food outlets are spread evenly across the entire SA2 as is the population, which may not be a valid assumption. Additionally, evidence-based thresholds used to collapse clusters into typologies were drawn from earlier studies set in different countries and contexts and may be different in relation to food offered, behaviours and health outcomes in metropolitan Melbourne, Australia. It is acknowledged that over the study period some change to SA2 boundaries may have occurred. However, between 2011 and 2016, approximately 95 % of SA2 boundaries remain effectively unchanged in Australia^(34)^. Nevertheless, for comparability, we used the year 2016 SA2 boundaries throughout. Finally, consistent with previous food retail environment studies, the SA2 ‘Melbourne’ (i.e. the CBD) was excluded due to the fact that food outlets in this area mainly service visitors^(36)^.

### Recommendations/implications for practice

The evidence to date suggests manipulating the food retail environment to support healthy food choices presents a potentially powerful opportunity to reduce the prevalence of obesity at the population level^(3,25)^. We present a key element for a comprehensive surveillance system which could provide evidence for planners, policy makers and interventionists, seeking to improve health through changing food retail environments. Implemented as part of a routine monitoring system, it would provide insight into drivers, trends and disparities in access to food resources, with particular emphasis on areas of low SEP and areas experiencing rapid population growth and expansion^(11,25,56)^. It is proposed that census measures of the entire food retail environment become the gold standard for future research, alongside measures guided by data-driven techniques that allow for identification of a broad range of food retail environment typologies. Additionally, it would be ideal for data to be provided by the relevant authority that regulates food retail (i.e. local governments) as these data are likely to have a higher level of accuracy^(57)^. These techniques are of international relevance for countries seeking to monitor, examine and identify emerging food retail environment trends and disparities alongside relationships with public health outcomes in greater detail. The evidence produced provides a source of data that could be linked to population health statistics to understand the relationships and trends over time, building the evidence base to support decision makers in favour of population health when challenged by the commercial interests of ‘big food’^(1)^. Further research using typologies and both child and adult BMI and dietary behaviours alongside other factors that may also influence access to food retail (e.g. income, employment and car ownership)^(58,59)^ are required to examine the impact of different food retail environments on populations according to socio-economic and geographic strata.

## Conclusion

We identified six food retail environment typologies representing relative healthy food availability and accessibility to the full spectrum of food outlets in Melbourne. All typologies were inherently unhealthy and pose potential increased risks to public health. Disparities across food retail environments were evident across areas of differing SEP and geographic locations and evolved over the eight-year study period. Communities living in low SEP areas and further from the CBD had largely low access to food outlets, and only a small proportion of these outlets were healthy. Whilst those living in areas of higher SEP and/or closer to the CBD were more likely to have high access to food outlets in general, and a marginally higher percentage of healthy outlets. This research provides new methods to understand the food retail environment and supports the need for food retail environment monitoring for the purposes of future research, strategic planning and enforcement of regulatory approaches to improve public health.

## Supplementary material

For supplementary material accompanying this paper visit https://doi.org/10.1017/S136898002200009X. click here to view supplementary material
