# Supplementary material for: Beyond food swamps and food deserts: exploring urban Australian food retail environment typologies
Source: Public Health Nutr. 2022 Jan 13;25(5):1140–52. doi: 10.1017/S136898002200009X (PMC9991784; doi:10.1017/S136898002200009X)
Supplement: Supplementary file 1 [file S136898002200009Xsup001.docx]

**Supplementary Table 1: Food outlet descriptions and Food Retail Environment Score for healthiness.**

| Food outlet type | Description | Health Score |
| --- | --- | --- |
| Fruiterer & greengrocer | Mainly engaged in the sale of fresh fruit and vegetables; including wholesale stores with direct to public sales | 10 |
| Fish shop | Mainly engaged in the sale of fresh seafood; including wholesale stores with direct to public sales and takeaway stores also providing a range of fresh seafood. | 9 |
| Poultry shop | Mainly engaged in the sale of fresh poultry; including wholesale stores with direct to public sales. | 9 |
| Butchery | Mainly engaged in the sale of fresh meat; including wholesale stores with direct to public sales. | 9 |
| Major Supermarket | Mainly engaged in the sale of groceries (fresh foods, canned and packaged foods, dry goods) of non-specialised (conventional) food lines. May contain a butcher or baker. Usually have 5 or more checkouts and a floor area over 1000 square meters. I.e. Woolworths, Coles, BI-LO, Franklins (no frills), ALDI. | 5 |
| Minor Supermarket | Mainly engaged in the sale of groceries (fresh foods, canned and packaged food, dry goods) of non-specialised (conventional) food lines. Usually have 4 or fewer checkouts and a floor area under 1000 square meters. E.g. Independent grocer or supermarket. | 5 |
| Specialty food stores – core foods | Mainly engaged in the sale of a limited line of specialised food such as a particular gourmet food that can be defined under core food. | 5 |
| Restaurant/café – franchise | E.g. franchise restaurants and cafes; mainly engaged in the preparation and sale of meals/snacks for consumption on the premises; table service provided; may sell alcohol with food; may provide takeaway services. | 0 |
| Restaurant/café – local independent | E.g. restaurant in a golf club, culture-based restaurant/café which is not a take-away such as Mexican, Thai, Chinese etc. ; mainly engaged in the preparation and sale of meals/snacks for consumption on the premises; table service provided; may also sell alcohol with food, may provide takeaway services. | 0 |
| Sandwich shop | Mainly engaged in the preparation of filled bread products like sandwiches or rolls. | 5 |
| Salad/sushi bar | Mainly engaged in the preparation of salads and sushi. | 5 |
| Delicatessen | Mainly engaged in the sale of specialty packaged or fresh products such as cured meats and sausage, pickled vegetables, dips, bread and olives; may also provide dine in meals. | 0 |
| Bakery | Mainly oriented towards bread, biscuits, pastries or other flour products with or without packaging. | 0 |
| General store | Mainly engaged in the sale of a limited line of groceries generally includes milk, bread and canned and packaged foods. | -5 |
| Specialty food store – extra foods | Mainly engaged in the sale of foods such as ice-creams, donuts, waffles, cakes etc. than can be defined under extra food. | -8 |
| Pub | E.g. pub within a bowling or lawn bowls park, pub inside a private gambling club; primarily engaged in selling alcoholic beverages where consumers can order and consume the alcoholic drinks and food on premises; can also be part of park or private club. | -5 |
| Take-away local independent | E.g. kebab, fish & chips, burger, chicken shops, local pizza, mainly engaged in the preparation and sale of meals/snacks that are ready for immediate consumption; table service not provided; meals can be eaten on site; taken away or delivered; shop is not a franchise. | -8 |
| Take-away franchise store | E.g. McDonalds, KFC, Subway; mainly engaged in the preparation and sale of meals (excludes donuts, drinks, ice-cream etc.)/snacks that area ready for immediate consumption; table service not provided; meal can be eaten on site, taken away or delivered; the food shop is a franchise/chain store with food being sold in specialised packaging. | -10 |

*Source: adapted from Moayyed et al. 2017^(22)^*; <https://onlinelibrary.wiley.com/doi/pdf/10.1111/1747-0080.12286>

**Supplementary File 2. Supporting information for Food Retail Environment Measures and the Relative Healthy Food Availability**

*Healthiness of the food retail environment (measured as RHFA):* Research indicates that people living in areas of Melbourne with RHFA >10%, have higher odds of purchasing healthier foods (fruit and vegetables), compared to households in areas with a RHFA <10%^(1)^. Another Australian study in the state of New South Wales, measured the proportion of unhealthy food outlets (fast-food and takeaway) relative to healthy outlets (grocery stores) within a road network buffer from participants home^(2)^. For analysis, in this study measures of the proporiton of unhealthy outlets were divided into quartiles (i.e., 1-25%; 25-50%; 50-75%; 75-99%) and three additional categories including zero food retail, 0% percent unhealthy and 100% unhealthy food outlets. Results indicated that compared to having no unhealthy outlets but having healthy food outlets within 3.2km from home, food retail environments comprising >25% unhealthy outlets within a 3.2km distance from home was associated with a BMI 1.37-1.19kg/m^2^ higher among adults^(2)^. Predicted mean BMI with 95% confidence intervals across quartiles illustrated that lower mean BMI among adults corresponded with lower levels of exposure to fast-food outlets both at 1.6km and particularly 3.2km^(2)^.

Guided by the evidence and earlier classification techniques, we proposed increasing healthiness is reflected in an increasing percentage of healthy outlets, with a RHFA measure of ≤25% representing the most unhealthy measure.

*Access and availability*. In the absence of a clear definition, good access to food resources in urban areas is considered to be a short driving distance (e.g. 5 minutes or 4km), or walking distance (e.g. 500m) from the residential home^(3-5)^. In Melbourne, planning standards for urban Growth Areas recommend the majority (at least 80%) of households be within 1km of a town centre, defined as an important community focal point with a mix of uses to meet the needs of the local community^(6)^. Guided by this recommendation, in this study we assumed a town centre with mixed use would likely house at least one each of ‘healthy’, ‘less healthy’ and ‘unhealthy’ food outlets. If the population is evenly located around the town centre on average there would be one of each type of outlet within 1km of most households. Therefore, access (as a measure of distance to travel) to outlets was considered ‘*low*’ when the average density of each type of store (healthy, unhealthy and less healthy) was less than one store per km^2^, ‘*moderate*’ if between 1 and 2 and ‘*high*’when the density per km^2^ was greater than two per km^2^. This last threshold was informed by the data, as many SA2s in Melbourne had access well in excess of what would be considered ‘*moderate*’ and for this reason needed differentiation.

Supermarkets are larger in floor size, quantity and type of products for sale compared to other food outlets. As such there are often fewer supermarkets, albeit operating at a larger scale than smaller independent retailers; and serving a greater proportion of the population (68% of food purchases were from supermarkets in 2019)^(7)^. The distance of 1.6km has been utilised in a number of Autralian studies as the catchment area for supermarkets, reflective of the neighbourhood area around a persons home and a 15-20 minute walking distance^(8-10)^. Preliminary evidence suggesting where communities do not have access to a supermarket within 1.6km it is more common to rely on public or private transport to obtain fresh produce^(9)^, introducing potential barriers to accessibility. The United States Department of Agriculture (USDA) also utlises a catchment area of 1.6km, defining food deserts as a low-income census tract within an urban area where at least 33 percent of the population cannot access a supermarket or large grocery store within one mile (1.6km) from home^(11)^. Guided by this threshold we defined access to supermarkets as ‘*low’* where density per km^2^ was below 0.625, ‘*moderate*’ when density of supermarkets was at least 0.625; and, ‘*high*’ where more than double (i.e., ≥ 1.25 supermarkets per km^2^) were reported.

**Supplementary Table 3. Cluster analysis results, summary statistics, classification and Food Retail Environment Typology.**

| Cluster | No | Food retail environment measure | Mean | SD | Min | Max | RHFA/Access | **Food** |
| --- | --- | --- | --- | --- | --- | --- | --- | --- |
|  | Obs |  |  |  |  |  | Classification | **Environment** |
|  |  |  |  |  |  |  |  | **Typology** |
|  |  | RHFA | 66.2 | 18 | 44.4 | 100 | >50% | **Low access – High % Healthy** |
| 13 | 81 | Healthy per km2 | 0.32 | 0.66 | 0 | 2.74 | Low |  |
|  |  | Unhealthy per km2 | 0.14 | 0.22 | 0 | 0.93 | Low |  |
|  |  | Less Healthy per km2 | 0.35 | 0.76 | 0 | 4 | Low |  |
|  |  | Supermarkets per km2 | 0.1 | 0.18 | 0 | 0.67 | Low |  |
| 8 | 228 | RHFA | 3.4 | 5.1 | 0 | 16.7 | ≤25% | **Low access – Low % healthy** |
|  |  | Healthy per km2 | 0.15 | 0.2 | 0 | 1.03 | Low |  |
|  |  | Unhealthy per km2 | 0.9 | 0.87 | 0 | 3.68 | Low |  |
|  |  | Less Healthy per km2 | 0.6 | 0.63 | 0 | 2.5 | Low |  |
|  |  | Supermarkets per km2 | 0.03 | 0.06 | 0 | 0.28 | Low |  |
| 15 | 384 | RHFA | 23.9 | 7.2 | 10 | 44.4 | ≤25% | **Moderate access – Low % healthy** |
|  |  | Healthy per km2 | 0.5 | 0.33 | 0 | 1.37 | Low |  |
|  |  | Unhealthy per km2 | 1.23 | 0.84 | 0.01 | 5.53 | Moderate |  |
|  |  | Less Healthy per km2 | 0.88 | 0.66 | 0 | 3.09 | Low |  |
|  |  | Supermarkets per km2 | 0.22 | 0.15 | 0 | 0.63 | Low |  |
| 9 | 13 | RHFA | 11.8 | 6 | 6.2 | 23.1 | ≤25% | **High Access – Low % healthy** |
|  |  | Healthy per km2 | 3.35 | 1.35 | 0.97 | 4.84 | High Access |  |
|  |  | Unhealthy per km2 | 9.68 | 2.34 | 6.36 | 13.85 | High Access |  |
|  |  | Less Healthy per km2 | 22.28 | 1.47 | 20 | 25.13 | High Access |  |
|  |  | Supermarkets per km2 | 0.81 | 0.34 | 0.32 | 1.29 | Moderate |  |
| 10 | 2 | RHFA | 11.2 | 1.2 | 10.3 | 12 | ≤25% | **High Access – Low % healthy** |
|  |  | Healthy per km2 | 4.44 | 0.79 | 3.89 | 5 | High Access |  |
|  |  | Unhealthy per km2 | 17.5 | 2.75 | 15.56 | 19.44 | High Access |  |
|  |  | Less Healthy per km2 | 66.94 | 2.75 | 65 | 68.89 | High Access |  |
|  |  | Supermarkets per km2 | 0.83 | 0.39 | 0.56 | 1.11 | Moderate |  |
| 3 | 2 | RHFA | 20.8 | 5.9 | 16.7 | 25 | ≤25% | **High Access – Low % healthy** |
|  |  | Healthy per km2 | 10 | 0 | 10 | 10 | High Access |  |
|  |  | Unhealthy per km2 | 15.6 | 0 | 15.6 | 15.6 | High Access |  |
|  |  | Less Healthy per km2 | 35.6 | 0 | 35.6 | 35.6 | High Access |  |
|  |  | Supermarkets per km2 | 2.6 | 0.85 | 2 | 3.2 | High Access |  |
| 4 | 3 | RHFA | 10.6 | 0.7 | 10 | 11.4 | ≤25% | **High Access – Low % healthy** |
|  |  | Healthy per km2 | 5.08 | 1.34 | 3.57 | 6.11 | High Access |  |
|  |  | Unhealthy per km2 | 23.97 | 1.43 | 22.78 | 25.56 | High Access |  |
|  |  | Less Healthy per km2 | 71.85 | 2.74 | 70 | 75 | High Access |  |
|  |  | Supermarkets per km2 | 0.98 | 0.23 | 0.71 | 1.11 | Moderate |  |
| 5 | 5 | RHFA | 15.8 | 4.3 | 12.5 | 22.2 | ≤25% | **High Access – Low % healthy** |
|  |  | Healthy per km2 | 3.1 | 0.44 | 2.5 | 3.75 | High Access |  |
|  |  | Unhealthy per km2 | 11.04 | 2.18 | 7.92 | 12.82 | High Access |  |
|  |  | Less Healthy per km2 | 28.63 | 0.82 | 27.69 | 29.74 | High Access |  |
|  |  | Supermarkets per km2 | 1.44 | 0.21 | 1.28 | 1.67 | Moderate |  |
| 6 | 2 | RHFA | 22.2 | 6.4 | 17.6 | 26.7 | ≤25% | **High Access – Low % healthy** |
|  |  | Healthy per km2 | 10 | 0.57 | 9.6 | 10.4 | High Access |  |
|  |  | Unhealthy per km2 | 17.8 | 0.28 | 17.6 | 18 | High Access |  |
|  |  | Less Healthy per km2 | 44.4 | 3.96 | 41.6 | 47.2 | High Access |  |
|  |  | Supermarkets per km2 | 2 | 0.57 | 1.6 | 2.4 | High Access |  |
| 7 | 13 | RHFA | 20.6 | 6.1 | 12.5 | 29 | ≤25% | **High Access – Low % healthy** |
|  |  | Healthy per km2 | 3.08 | 0.87 | 2.14 | 4.69 | High Access |  |
|  |  | Unhealthy per km2 | 10.76 | 1.49 | 9.03 | 14.48 | High Access |  |
|  |  | Less Healthy per km2 | 16.63 | 2.29 | 13.21 | 19.66 | High Access |  |
|  |  | Supermarkets per km2 | 1.45 | 0.41 | 1.03 | 2.19 | Good |  |
| 16 | 3 | RHFA | 6.7 | 0.9 | 5.9 | 7.7 | ≤25% | **High Access – Low % healthy** |
|  |  | Healthy per km2 | 7.69 | 1.33 | 6.15 | 8.46 | High Access |  |
|  |  | Unhealthy per km2 | 18.21 | 2.35 | 16.15 | 20.77 | High Access |  |
|  |  | Less Healthy per km2 | 25.38 | 5.04 | 20.77 | 30.77 | High Access |  |
|  |  | Supermarkets per km2 | 0.77 | 0 | 0.77 | 0.77 | Moderate |  |
| 17 | 6 | RHFA | 20.5 | 6 | 12.2 | 29.4 | ≤25% | **High Access – Low % healthy** |
|  |  | Healthy per km2 | 4.9 | 0.68 | 4.14 | 6 | High Access |  |
|  |  | Unhealthy per km2 | 14.93 | 1.95 | 12.1 | 16.8 | High Access |  |
|  |  | Less Healthy per km2 | 27.08 | 3.5 | 22.19 | 31.03 | High Access |  |
|  |  | Supermarkets per km2 | 1.79 | 0.42 | 1.03 | 2.19 | Moderate |  |
| 19 | 3 | RHFA | 16.5 | 1.7 | 14.8 | 18.2 | ≤25% | **High Access – Low % healthy** |
|  |  | Healthy per km2 | 6.19 | 1.49 | 5 | 7.86 | High Access |  |
|  |  | Unhealthy per km2 | 29.76 | 2.51 | 27.14 | 32.14 | High Access |  |
|  |  | Less Healthy per km2 | 75 | 2.58 | 72.14 | 77.14 | High Access |  |
|  |  | Supermarkets per km2 | 2.14 | 0 | 2.14 | 2.14 | Very good |  |
| 20 | 127 | RHFA | 19.8 | 8.8 | 0 | 50 | ≤25% | **High Access – Low % healthy** |
|  |  | Healthy per km2 | 1.38 | 0.56 | 0 | 2.74 | Moderate |  |
|  |  | Unhealthy per km2 | 3.54 | 1.18 | 0.34 | 6.6 | High Access |  |
|  |  | Less Healthy per km2 | 4.67 | 1.78 | 1.05 | 10.43 | High Access |  |
|  |  | Supermarkets per km2 | 0.38 | 0.23 | 0 | 0.83 | Low |  |
| 11 | 29 | RHFA | 32 | 11.6 | 16.7 | 62.5 | >25% | **High Access – Moderate % healthy** |
|  |  | Healthy per km2 | 2.83 | 1.28 | 0.83 | 4.9 | High Access |  |
|  |  | Unhealthy per km2 | 4.94 | 1.62 | 2.5 | 8.43 | High Access |  |
|  |  | Less Healthy per km2 | 13.59 | 2.32 | 10 | 18.28 | High Access |  |
|  |  | Supermarkets per km2 | 1.22 | 0.47 | 0.34 | 2.17 | Moderate |  |
| 12 | 95 | RHFA | 30.3 | 8.1 | 15.9 | 50 | >25% | **High Access – Moderate % healthy** |
|  |  | Healthy per km2 | 3.12 | 0.94 | 1.18 | 5.71 | High Access |  |
|  |  | Unhealthy per km2 | 4.99 | 1.52 | 2.5 | 10 | High Access |  |
|  |  | Less Healthy per km2 | 6.68 | 1.71 | 2.69 | 10.23 | High Access |  |
|  |  | Supermarkets per km2 | 1.22 | 0.42 | 0.47 | 2.86 | Moderate |  |
| 14 | 12 | RHFA | 36.2 | 5.3 | 26.4 | 45.4 | >25% | **High Access – Moderate % healthy** |
|  |  | Healthy per km2 | 6.59 | 1.11 | 4.29 | 7.92 | High Access |  |
|  |  | Unhealthy per km2 | 7.93 | 1.1 | 5.83 | 10 | High Access |  |
|  |  | Less Healthy per km2 | 13.97 | 2 | 10.83 | 16.86 | High Access |  |
|  |  | Supermarkets per km2 | 2.4 | 0.43 | 1.67 | 3.2 | High Access |  |
| 1 | 2 | RHFA | 27.8 | 1.6 | 26.7 | 28.9 | >25% | **High Access – Moderate % healthy** |
|  |  | Healthy/ km2 | 10.6 | 1.98 | 9.2 | 12 | High Access |  |
|  |  | Unhealthy per km2 | 17 | 0.28 | 16.8 | 17.2 | High Access |  |
|  |  | Less Healthy per km2 | 37.6 | 0.57 | 37.2 | 38 | High Access |  |
|  |  | Supermarkets per km2 | 1.2 | 0.57 | 0.8 | 1.6 | Moderate |  |
| 2 | 1 | RHFA | 30.6 | . | 30.6 | 30.6 | >25% | **High Access – Moderate % healthy** |
|  |  | Healthy per km2 | 14 | . | 14 | 14 | High Access |  |
|  |  | Unhealthy per km2 | 17.6 | . | 17.6 | 17.6 | High Access |  |
|  |  | Less Healthy per km2 | 43.2 | . | 43.2 | 43.2 | High Access |  |
|  |  | Supermarkets per km2 | 2.8 | . | 2.8 | 2.8 | High Access |  |
| 18 | 164 | RHFA | 32.5 | 8.9 | 15.4 | 75 | >25% | **High Access – Moderate % healthy** |
|  |  | Healthy per km2 | 1.48 | 0.46 | 0.59 | 2.86 | Moderate |  |
|  |  | Unhealthy per km2 | 2.36 | 0.81 | 0.67 | 4.6 | High Access |  |
|  |  | Less Healthy per km2 | 2.24 | 1.04 | 0 | 5.48 | High Access |  |
|  |  | Supermarkets per km2 | 0.65 | 0.22 | 0.25 | 1.61 | Moderate |  |

*Represents Statistical Area 2’s with zero food retail outlets.

*No obs: number of observations

RHFA %: proportion as a percentage of healthy food retail outlets from the total number of food retail outlets within each SA2.

Per km2: per square kilometre.

SA2: Statistical Area 2: medium-sized general purpose areas representing geographical areas where community interact together socially and economically^(12)^

**Supplementary File 4. Statistical Area 2 location and food retail environment typologies in Greater Melbourne over time** **grouped by distance from the Central Business District**

| 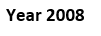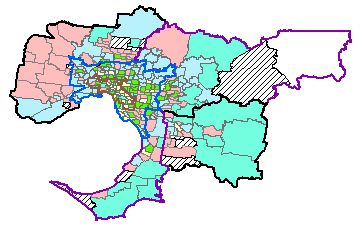 | 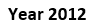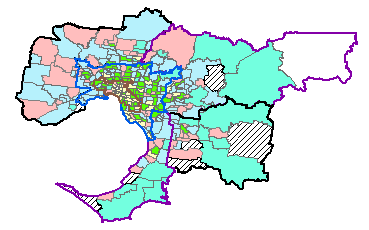 |  |
| --- | --- | --- |
| 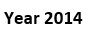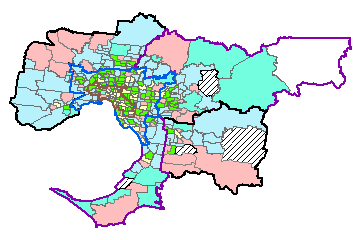 | 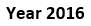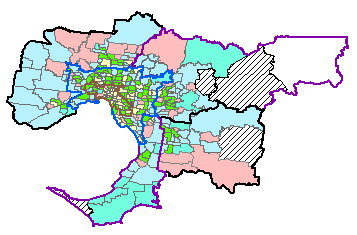 | *Shaded white: one Statistical Area 2 is excluded as it represents a Statistical Area 2 not included within Metropolitan Melbourne as classified by the Australian Bureau of Statistcs^(12)^ but sits within a local government area within metro Melbourne.  LGA-Ring: represents the grouping of local government areas based on geographical distance form the Cetral Buisiness District; Growth Areas represent designated local government areas expected to house a large proportion of urban growth. |
| **Food Retail Environment Typology** | **LGA-Ring** |  |
| **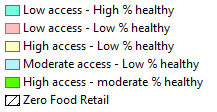** | 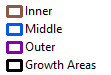 |  |
|  | |  |

**Supplementary Table 5. Food retail environment typology prevalence across area-level socioeconomic position within each year in Greater Melbourne**

|  | **SEIFA-IRSAD Quartiles** | | | | | | | | | |
| --- | --- | --- | --- | --- | --- | --- | --- | --- | --- | --- |
|  | **Q1 (lowest SEP)** | | **Q2** | | **Q3** | | **Q4 (highest SEP)** | | **Total** | **% of Total** |
| **Food Retail Environment Typology** | **No. SA2s** | **% of**  **each typology** | **No. SA2s** | **% of**  **each typology** | **No. SA2s** | **% of**  **each typology** | **No. SA2s** | **% of**  **each typology** | **No. SA2s** | **%** |
| **Year : 2008** | | | | | | | | | | |
| **Zero food retail** | 2 | 14.3 | 4 | 28.6 | 5 | 35.7 | 3 | 21.4 | 14 | 4.7 |
| **Low access - High % healthy** | 0 | 0.0 | 6 | 27.3 | 7 | 31.8 | 9 | 40.9 | 22 | 7.4 |
| **Low access - Low % healthy** | 19 | 24.4 | 19 | 24.4 | 27 | 34.6 | 13 | 16.7 | 78 | 26.3 |
| **Moderate access – Low % healthy** | 19 | 22.3 | 13 | 15.3 | 25 | 29.4 | 28 | 32.9 | 85 | 28.6 |
| **High access – Low % healthy** | 3 | 7.7 | 4 | 10.3 | 10 | 25.6 | 22 | 56.4 | 39 | 13.1 |
| **High Access – Moderate % healthy** | 9 | 15.2 | 7 | 11.9 | 16 | 27.1 | 27 | 45.8 | 59 | 19.9 |
| **Total** | 52 | 17.5 | 53 | 17.8 | 90 | 30.3 | 102 | 34.3 | 297 | 100.0 |
| **Year : 2012** | | | | | | | | | | |
| **Zero food retail** | 0 | 0.0 | 2 | 28.6 | 3 | 42.9 | 2 | 28.6 | 7 | 2.4 |
| **Low access - High % healthy** | 1 | 4.8 | 6 | 28.6 | 5 | 23.8 | 9 | 42.9 | 21 | 7.1 |
| **Low access - Low % healthy** | 15 | 25.9 | 13 | 22.4 | 18 | 31.0 | 12 | 20.7 | 58 | 19.5 |
| **Moderate access – Low % healthy** | 21 | 21.4 | 18 | 18.4 | 34 | 34.7 | 25 | 25.5 | 98 | 33.0 |
| **High access – Low % healthy** | 2 | 4.4 | 7 | 15.6 | 12 | 26.7 | 24 | 53.3 | 45 | 15.1 |
| **High Access – Moderate % healthy** | 13 | 19.1 | 7 | 10.3 | 18 | 26.5 | 30 | 44.1 | 68 | 22.9 |
| **Total** | 52 | 17.5 | 53 | 17.8 | 90 | 30.3 | 102 | 34.3 | 297 | 100.0 |
| **Year : 2014** | | | | | | | | | | |
| **Zero food retail** | 0 | 0.0 | 0 | 0.0 | 3 | 75.0 | 1 | 25.0 | 4 | 1.3 |
| **Low access - High % healthy** | 2 | 11.1 | 4 | 22.2 | 5 | 27.8 | 7 | 38.9 | 18 | 6.1 |
| **Low access - Low % healthy** | 8 | 17.8 | 10 | 22.2 | 16 | 35.6 | 11 | 24.4 | 45 | 15.1 |
| **Moderate access – Low % healthy** | 17 | 17.0 | 27 | 27.0 | 35 | 35.0 | 21 | 21.0 | 100 | 33.7 |
| **High access – Low % healthy** | 3 | 7.0 | 3 | 7.0 | 9 | 20.9 | 28 | 65.1 | 43 | 14.5 |
| **High Access – Moderate % healthy** | 17 | 19.5 | 8 | 9.2 | 25 | 28.7 | 37 | 42.5 | 87 | 29.3 |
| **Total** | 47 | 15.8 | 52 | 17.5 | 93 | 31.3 | 105 | 35.3 | 297 | 100.0 |
| **Year : 2016** | | | | | | | | | | |
| **Zero food retail** | 1 | 25.0 | 1 | 25.0 | 2 | 50.0 | 0 | 0.0 | 4 | 1.3 |
| **Low access - High % healthy** | 1 | 6.2 | 5 | 31.2 | 2 | 12.5 | 8 | 50.0 | 16 | 5.4 |
| **Low access - Low % healthy** | 6 | 14.6 | 9 | 21.9 | 16 | 39.0 | 10 | 24.4 | 41 | 13.8 |
| **Moderate access – Low % healthy** | 14 | 14.3 | 23 | 23.5 | 41 | 41.8 | 20 | 20.4 | 98 | 33.0 |
| **High access – Low % healthy** | 5 | 10.2 | 3 | 6.1 | 12 | 24.5 | 29 | 59.2 | 49 | 16.5 |
| **High Access – Moderate % healthy** | 20 | 22.5 | 11 | 12.4 | 20 | 22.5 | 38 | 42.7 | 89 | 30.0 |
| **Total** | 47 | 15.8 | 52 | 17.5 | 93 | 31.3 | 105 | 35.3 | 297 | 100.0 |

SEIFA-IRSAD: Socioeconomic Index for Areas – Index of Relative Socioeconomic Advantage and Disadvantage

SEP: Socioeconomic position Q: Quartile CBD: Central business district

*SA2: Statistical area 2: medium-sized general purpose areas representing geographical areas where community interact together socially and economically^(12)^

1. Mason KE, Bentley RJ, Kavanagh AM. Fruit and vegetable purchasing and the relative density of healthy and unhealthy food stores: evidence from an Australian multilevel study. Journal Of Epidemiology And Community Health. 2013;67(3):231-6.

2. Feng X, Astell-Burt T, Badland H, et al. Modest ratios of fast food outlets to supermarkets and green grocers are associated with higher body mass index: Longitudinal analysis of a sample of 15,229 Australians aged 45 years and older in the Australian National Liveability Study. Health & Place. 2018;49:101-10.

3. Luan H, Law J, Quick M. Identifying food deserts and swamps based on relative healthy food access: a spatio-temporal Bayesian approach. International Journal Of Health Geographics. 2015;14:37-.

4. O'Dwyer LA, Coveney J. Scoping supermarket availability and accessibility by socio-economic status in Adelaide. Health Promotion Journal Of Australia: Official Journal Of Australian Association Of Health Promotion Professionals. 2006;17(3):240-6.

5. Needham, C.; Sacks, G.; Orellana, L, et al. A systematic review of the Australian food retail environment: Characteristics, variation by geographic area, socioeconomic position and associations with diet and obesity. Obesity Reviews. 2019:1-22.

6. GAA. Precinct Structure Planning Guidelines Part Two: Preparing the pricnct strucutre plan. Growth Areas Authority. Melbourne, Australia: Victorian State Government; 2013.

7. Crothers L. Australia Retail Foods : Retail Food Sector Report 2019. USDA Foreign Agricultural Service : Global Agricultural Information Network; 2019.

8. Bivoltsis A, Trapp G, Knuiman M, et al. The evolution of local food environments within established neighbourhoods and new developments in Perth, Western Australia. Health & Place. 2019;57:204-17.

9. Astell-Burt T, Feng X. Geographic inequity in healthy food environment and type 2 diabetes: can we please turn off the tap? The Medical Journal Of Australia. 2015;203(6):246-8.e1.

10. Giles-Corti B, Timperio A, Cutt H, et al. Development of a reliable measure of walking within and outside the local neighborhood: RESIDE's Neighborhood Physical Activity Questionnaire. Preventive Medicine: An International Journal Devoted to Practice and Theory. 2006;42(6):455-9.

11. USDA. Mapping Food Deserts in the United States. United States Department of Agriculture ERS. United States of Amercia: Amber Waves; 2011.

12. ABS. 1270.0.55.001 - Australian Statistical Geography Standard (ADGS): Volume 1 - Main Structure and Greater Capital City Statistical Areas, July 2016: Australian Federal Government; 2016 [updated July 2016; cited 2020 January 4]. Available from: <https://www.abs.gov.au/ausstats/abs@.nsf/Lookup/by%20Subject/1270.0.55.001~July%202016~Main%20Features~Statistical%20Area%20Level%202%20(SA2)~10014>.
